# La supervivencia por sobre el ideal de maternidad: experiencias de duelo migratorio de las madres migrantes en Chile

**DOI:** 10.18294/sc.2023.4649

**Published:** 2023-12-18

**Authors:** Lucia Odette Castillo Lobos, Luis Patricio Contreras Vásquez, Elizabeth Yenny Hermosilla Aldea

**Affiliations:** 1 Doctora en Estudios Americanos mención en Estudios Sociales y Políticos. Académica Escuela de Enfermería, Universidad de Santiago de Chile, Santiago, Chile. lucia.castillo@usach.cl Universidad de Santiago de Chile Universidad de Santiago de Chile Santiago Chile lucia.castillo@usach.cl; 2 Magíster en Salud Mental. Académico, Escuela de Enfermería, Universidad de Santiago de Chile, Santiago, Chile. luis.contreras.v@usach.cl Universidad de Santiago de Chile Escuela de Enfermería Universidad de Santiago de Chile Santiago Chile luis.contreras.v@usach.cl; 3 Magíster en Educación Superior e Investigación Aplicada. Docente, Escuela de Enfermería, Universidad de Santiago de Chile, Santiago, Chile. elizabeth.hermosilla@usach.cl Universidad de Santiago de Chile Escuela de Enfermería Universidad de Santiago de Chile Santiago Chile elizabeth.hermosilla@usach.cl

**Keywords:** Migración Humana, Duelo, Mujeres, Chile, Human Migration, Grief, Women, Chile

## Abstract

Este artículo da cuenta de una aproximación al fenómeno de duelo migratorio de las madres migrantes en Chile. Entre 2021 y 2022 se realizó un estudio cualitativo y exploratorio sustentado en el interaccionismo simbólico, en el que se realizaron entrevistas semiestructuradas a 39 madres migrantes: 18 de origen venezolano, 11 peruano y 9 haitiano. A partir del análisis de contenido temático y de la teorización, se obtuvieron tres categorías de análisis: 1) la pérdida de los vínculos y la lejanía con el origen, 2) expectativas versus realidad y 3) validando sobreesfuerzos. Las madres migrantes manifiestan el duelo migratorio con distintos matices según su país de origen e imprimen en cada una de sus experiencias, en mayor o menor grado, el desarraigo y la ausencia de redes de apoyo como principal detonante de la tristeza que viven día a día. Esta tristeza se acrecienta al negociar su ideal de criar personalmente a sus hijos e hijas en pro de la subsistencia material para alcanzar la ansiada vida mejor, para la cual validan el sobreesfuerzo como estrategia de superación. Estas presiones traen consigo consecuencias psíquicas y físicas que impiden la elaboración del duelo migratorio, comprometiendo su salud mental y la de sus hijos e hijas.

## Abstract (en)

This article presents an approach to the phenomenon of migratory mourning of migrant mothers in Chile. Between 2021 and 2022, a qualitative exploratory study based on symbolic interactionism was carried out, in which semi-structured interviews were conducted with 39 migrant mothers of Venezuelan (18), Peruvian (11) and Haitian (9) origin. Based on a theoretically-informed thematic analysis, three analytical categories emerged: 1) the loss of social ties and distance to their place of origin; 2) expectations versus reality; and 3) the validation of overexertion. Migrant mothers express migratory mourning with varying levels of nuance depending on their country of origin, and to a greater or lesser degree signify their experiences in terms of uprooting and the absence of support networks, which they identify as the main cause of the sadness they experience on a daily basis. This sadness increases when they negotiate their ideals regarding the upbringing of their sons and daughters in favor of material subsistence in order to achieve the goals of a better life, and for which they justify overexertion as a strategy for improvement. These pressures bring with them psychological and physical consequences and interfere with their processes of migratory mourning, compromising their mental health and that of their children.

## INTRODUCCIÓN

Joseba Achotegui^1^ señala que la migración, como evento vital, es concebida por quienes migran como un puente dibujado por las expectativas de acceder a una mejora de oportunidades sociales y horizontes vitales^1^. Según la misma autora, todos los individuos de la especie humana descenderían de quienes ya han migrado a lo largo del proceso evolutivo humano, por lo tanto, los hombres y las mujeres ya dispondrían de los recursos necesarios para migrar^1^. Sin embargo, el actual carácter dinámico de los fenómenos migratorios, los cambios en el patrón migratorio y sus diversas motivaciones e impactos sociales, económicos y culturales dibujan una serie de situaciones estresantes para los grupos migrantes que los obligan a desplegar variadas estrategias para su afrontamiento y que, por tanto, tensionan este planteamiento. 

A nivel latinoamericano, destaca el fenómeno migratorio chileno que, desde la década de 1990 oscila entre ser un país expulsor de población, sobre todo a Argentina y Europa, a experimentar un aumento de la población inmigrante proveniente preferentemente desde Perú. Ya en la década de 2010, el país pasó a ser escenario de un crecimiento exponencial de la inmigración en el período, alcanzando una tasa del 4,9% anual, seguido de México (4,2%) y Brasil (3,8%)^2^, lo que se exacerbó a partir de la segunda mitad de la misma década como fruto de la crisis venezolana y haitiana^2^ y que, al mismo tiempo, han coincidido con la estabilidad política y económica en Chile y han erigido al país como destino de población migrante latinoamericana y del caribe en busca de oportunidades laborales y económicas^3^^,^^4^^,^^5^, pero también, como destino de reunificación familiar^6^^,^^7^^) .^

En 2020, las personas extranjeras residentes habituales en Chile correspondían a 1.462.103 personas distribuidas a nivel nacional, con una proporción mayor en la Región Metropolitana (61,9%), Antofagasta (7,0%), Tarapacá (5,9%) y Valparaíso (6,6 %)^8^. En relación con los países de origen, para el mismo año, la comunidad venezolana alcanzaba un 30,7% del total de personas extranjeras residentes, siendo la de mayor presencia. En segundo lugar, se ubicaba la comunidad peruana con un 16,3% y, en tercer lugar, la comunidad haitiana con un 12,5%^8^.

El actual proceso migratorio hacia Chile, además de su alto dinamismo, mantiene una tendencia similar a la de otros países receptores, con relación a su concentración eminentemente urbana, el origen centro y sudamericano de la población inmigrante, su integración laboral segmentada en trabajos más precarios, propios de los sectores más vulnerables socialmente, y por la tendencia a la feminización de algunas comunidades migrantes^9^^,^^10^^,^^11^^,^^12^^,^^13^. Esta tendencia a la feminización y la reunificación familiar implica migración de infantes y aumento del número de nacimientos en el país^14^^,^^15^, quienes se espera sean usuarios y usuarias del sistema preescolar y público de salud en Chile y que se asentarán en una sociedad chilena de destino, que constituye para ellas un espacio social de la diferencia y la no pertenencia social y cultural^16^. En particular, las madres inmigrantes en Chile enfrentan obstáculos tales como la integración a una cultura y un idioma diferentes, la separación de sus seres queridos y la necesidad de dar subsistencia a sus familias en un ambiente desconocido y que ofrece precarias condiciones de subsistencia en el contexto de una sociedad chilena que tiende a ser hegemónica, discriminatoria, xenófoba y racista^14^. 

En esta cotidianidad se funden las experiencias de las madres con relación a su país de origen, su trayectoria migratoria, la existencia de redes de apoyo, el nivel socioeconómico alcanzado en la sociedad de destino y la relación establecida con sus superestructuras sociales, y en las que además se imprimen sus acervos culturales, los resabios de dolorosas vivencias de vulneración social, la precariedad de sus espacios de subsistencia y la discriminación que, algunas de ellas, experimentan día a día y que surten efectos en la salud mental de las madres que, a su vez, se transmite a sus hijos e hijas a través de un vínculo sanguíneo fortalecido por la cotidianidad de la crianza en migración y desarraigo. 

Desde la experiencia internacional es posible identificar la precariedad en los espacios de vida de la población migrante como una problemática transversal manifestada, principalmente, en la ausencia de redes de apoyo familiares para abordar la crianza de los niños y las niñas en una nueva realidad vivida en la que aflora una percepción de “carga” de responsabilidad parental a causa de la migración, traducida en miedo, preocupación, tristeza, soledad y agotamiento de los padres^17^. Por otro lado, desde la perspectiva de los contextos en que las madres migrantes sitúan el cuidado de sus hijos e hijas, es importante señalar el papel de la transnacionalidad en este fenómeno, en el que muchas mujeres migrantes comparten la responsabilidad de garantizar la reproducción social y mantener los vínculos de parentesco más allá de las fronteras chilenas, al asumir la responsabilidad como madres, hermanas o hijas, y de otras redes de parentesco a través del envío, a su país de origen, de remesas, bienes, cuidados, afectos^18^ y también remesas sociales, ideas, valores y creencias, que dan cuenta del carácter político-económico y privado del cuidado transnacional vinculado a la familia y el hogar^19^. 

Sustentados en estos antecedentes, Martínez y García^20^ ya definen la crianza migrante como expresión de una “maternidad difícil”^20^, dada la situación de riesgo y estrés que conlleva para la madre y sus hijos e hijas, y que, además, puede comprometer la calidad del cuidado materno e influir en la salud mental y física de las niñeces tanto en la infancia como en la vida adulta^19^ y que se acrecientan en condiciones de precariedad y estrés materno^21^. Desde ese lugar, para velar por la salud mental de niños y niñas de manera directa, también es necesaria la protección de sus cuidadores^22^, sobre la base de una amplia literatura que señala que la salud mental de las madres se relaciona con la salud mental de los hijos^23^. Desde la perspectiva de los niños y las niñas, el análisis sociocultural de temáticas vinculadas a acciones en salud en el contexto de crianza de hijos e hijas de madres migrantes encuentra mayor asidero, en tanto la evidencia releva la influencia de las diferencias culturales y ambientales en el desarrollo integral de niños y niñas^24^^).^ Otros estudios también indican que la mejora de las condiciones de habitabilidad y educación de las madres surten efectos positivos en la salud de sus hijos e hijas^25^.

Como producto de estas experiencias, algunas madres migrantes vivencian el llamado duelo migratorio, que involucra a aquellas experiencias emocionales producto del desarraigo y pérdida de conexiones familiares y comunitarias para responder a la necesidad de integrarse a un nuevo ambiente cultural, y que se da bajo una sensación de aislamiento y soledad. Algunas fuentes refieren que el duelo de las madres migrantes iría más allá: estas mujeres vivirían un doble duelo por la pérdida de sus redes de apoyo para la asistencia en la crianza de sus hijos e hijas, como las propias, producto de su movilidad, lo que podría generar que posterguen sus proyectos personales para priorizar su rol de cuidadoras^22^. Respecto a este punto, existe evidencia que indica que, pese a la permanente presencia de las mujeres en los fenómenos migratorios, su salud mental no ha sido abordada de forma específica. Desde ese lugar, si bien el síndrome de Ulises evalúa un conjunto de vulnerabilidades y estresores en ausencia de una perspectiva de género, pese a que la salud mental de las mujeres en general parece estar relacionada con roles y estereotipos de género. Estas situaciones no permiten que la salud mental de las mujeres migrantes se evalúe de forma integral, considerando sus perspectivas individuales, sociales y culturales^26^. En el caso de las madres inmigrantes en Chile, el duelo migratorio puede ser particularmente agudo debido a la importancia cultural que se otorga a la maternidad y la crianza en un país que tensiona el concepto ideal de crianza aprendido en su país de origen y que frustra sus expectativas de ser una buena madre dentro de su proyecto migratorio. Estas tensiones se ven profundizadas ante la necesidad de integrarse a una sociedad de destino que exhibe prácticas culturales distintas y que, consecuentemente, puede hacerlas sentir aisladas, solas, ansiosas y deprimidas, debido a la discriminación, el racismo y la precariedad en que habitan. 

Por otro lado, la falta de acceso a servicios básicos, como la atención médica y educación, puede exacerbar el estrés y la ansiedad asociados con la adaptación a un nuevo ambiente, y que redunda en el estrés parental por migración. Respecto a este punto, una revisión sistemática realizada por Fellmeth^27^ concluyó que una de cada tres mujeres que migran desde países con bajos recursos experimenta síntomas compatibles con depresión perinatal^27^. Complementariamente, un estudio cuantitativo realizado en Chile, que tuvo como objetivo estudiar el estrés parental por migración en madres migrantes, concluyó que aquellas madres que tuvieron su primer hijo en el lugar de origen mostraron un mayor nivel de estrés parental por migración en comparación a aquellas mujeres que tuvieron a su primer hijo en Chile, dada la presión de adaptarse con mayor velocidad al contexto chileno en ausencia de redes de apoyo^28^. Por otro lado, un estudio de Bernales, Cabieses y McIntyre^29^evidenció las dificultades en cuanto al bienestar psicosocial y emocional al que de manera transversal estaban expuestos padres y madres migrantes viviendo en Chile^29^. Otros estudios realizados en el país dan cuenta de que los estresores psicosociales que afectan a las mujeres migrantes que llegan a Chile no se verían reflejados en cuestionarios de salud mental, debido a la “paradoja del migrante”, situación en la que los sujetos migrantes, pese a sufrir múltiples factores de estrés, presentan mejores niveles de salud mental que las personas en general, explicado por la resiliencia, esperanza y mirada optimista de los migrantes hacia el futuro^30^, y la fe y religiosidad propia de las mujeres migrantes latinas como factor protector frente al estrés de la migración conlleva^31^.** **Sin embargo, esta salud física y mental de las personas migrantes tendería a decaer con el tiempo con relación a factores relacionados con el estatus legal y los procesos de racialización^32^.

Desde ese lugar, ampliar el concepto de salud y salud mental a un completo estadio de bienestar significa comprender la salud como un concepto abstracto y relativo que posee una importante carga de subjetividad, y que guarda relación con factores económicos, que llevan a minimizar la desigualdad social que hace referencia a la calidad de vida y a la satisfacción integral de las necesidades humanas^33^^,^^34^. Es así como a la luz del “modelo de dos continuos” la salud mental se erige como un estado de bienestar independiente de la presencia o no de una enfermedad diagnosticada^35^, en que las presiones socioeconómicas, marginación social, violencia, exposición a violaciones de los derechos humanos y la desvinculación con redes de apoyo social e institucional, comprometen la salud mental de quienes migran, incluso en ausencia de una enfermedad mental diagnosticada. Cabe aclarar que, en sí mismo, el fenómeno migratorio no constituye una causa directa de enfermedad mental^1^, sin embargo, los espacios de subsistencia en que se asienta la población migrante, en efecto constituyen un factor de riesgo para la salud mental, lo que revela la importancia de mejorar las condiciones de integración de la población migrante a la sociedad chilena, dados los beneficios descritos en la salud mental y que, a su vez, se transmiten a los niños y las niñas. 

Con el objetivo de analizar las vivencias de duelo migratorio de las mujeres migrantes, en ocasión del ejercicio de su rol como madres que crían en Chile, se han formulado las siguientes preguntas de investigación: ¿Cómo se expresa este duelo migratorio en la cotidianidad de la integración de las madres migrantes a la sociedad chilena? ¿Cómo se resignifica este duelo migratorio en el ejercicio de su rol materno? Se plantea responder a estas incertidumbres a partir del marco interpretativo del estrés por duelo migratorio que establece un fuerte vínculo entre duelo y estrés psicológico producto de la migración^1^. Este concepto desarrollado por Joseba Achotegui^36^, indica que este tipo de duelo ocurre en personas migrantes que experimentan eventos vitales críticos en migración, tales como ser víctimas de políticas de exclusión y de explotación laboral, vivenciar carencias sanitarias y de vivienda, o por problemas de personalidad del sujeto migrante, al punto de desencadenar una desorganización psicológica^36^. 

Como todo tipo de duelo, el duelo migratorio presenta fases reconocibles, a saber: la negación como etapa en que los individuos no aceptan la nueva vida ofrecida en la sociedad de destino; una segunda etapa, en la que se identifica la resistencia, manifestada por quejas y protestas frente el esfuerzo y obstáculos que supone la migración; una tercera etapa, en la que los sujetos migrantes aceptan las nuevas formas de vida de la sociedad de destino; y en la última etapa de este duelo migratorio, identificada con la restitución, tiene efecto la reconciliación afectiva con lo que se ha dejado atrás y con la nueva vida en migración^36^. Como consecuencia, este duelo migratorio puede evolucionar hacia tres instancias: por una parte, hacia un “duelo simple”, en que las condiciones individuales y ambientales permiten la resolución del duelo de manera eficaz logrando la integración; hacia un “duelo complicado”, en el que las nuevas condiciones de vida entorpecen la resolución del duelo, o hacia un “duelo extremo”, en el que la magnitud de los obstáculos para la integración social impide la resolución del duelo^1^. Este duelo extremo es conocido como el “síndrome de Ulises”, llamado así en referencia al relato mítico contenido en *La Odisea*, donde su protagonista Odiseo (o Ulises), al finalizar la guerra de Troya, emprende el viaje de vuelta a su tierra natal enfrentando una serie de dificultades designadas por los dioses^37^. En este cuadro clínico de carácter crónico y de origen multifactorial dentro del proceso migratorio, las personas experimentan un extremo dolor psíquico con signos ansiosos, depresivos, somatomorfos y/o confusionales, asociados a sentimientos de soledad, desesperación, indefensión, miedo, desprotección y pobreza^1^. 

### La salud mental de las personas migrantes

Según Erich Fromm, el ser humano ha emergido del reino animal, trascendiendo a la adaptación instintiva y a la naturaleza en el desarrollo de la razón. Según su planteamiento, “el hombre debe sobrevivir no solo física, sino que también psíquicamente, en tanto necesita conservar cierto equilibrio psíquico para no perder la capacidad de funcionar. Así, el mantenimiento de un equilibrio psíquico alcanzaría la misma relevancia que el equilibrio físico, con el afán construir su sentido de identidad^38^. La razón y la capacidad de abstracción condicionarían al ser humano para responder a necesidades específicas, que deben ser satisfechas de manera insoslayable para su autopreservación desde lo psíquico, y si bien no necesariamente son percibidas de manera directa por las personas, sus actos las ponen en evidencia y las explican^39^. A partir de estas necesidades, Fromm desarrollo su conceptualización de la salud mental, al grado en que estas necesidades sean adecuadamente satisfechas, ajustadas al contexto ambiental, histórico y social, y que determinan el grado de salud mental que la persona puede alcanzar. 

Con este antecedente, Fromm^39^ define las siguientes necesidades:

*De vinculo*: el ser humano necesita crear nuevos lazos con el mundo a través del establecimiento de relaciones vinculantes con otras personas, y la manera en que busca establecer estos vínculos dependerá de factores históricos, culturales, familiares, políticos y socioeconómicos.

*De marco de orientación*: el ser humano busca maneras para orientar sus decisiones. Con su razonamiento distingue dos planos para su marco de orientación: el primero es existencial, un marco de referencia que determine la conceptualización de lo sano y lo insano, mientras el segundo plano define la calidad de vida, la comparación entre presencia o ausencia de bienestar.

*De poder entregarse a algo*: a través del planteamiento de una meta que oriente su energía para actuar y dar sentido a la propia vida y conectar los vínculos con la existencia.

*De arraigo*: se sustenta en el vínculo primordial con la madre y el origen, la cual es necesaria para encontrar un espacio personal propio para sentirse como en casa, enraizarse y crear sus nuevos vínculos.

*De identidad*: los individuos se sienten seguros de sus propios actos reafirmando su propia individualidad y su identificación con elementos de comunidad, tales como la raza, la nacionalidad, el idioma, la religión y las tradiciones.

*De trascendencia*: el ser humano expresa su necesidad de trascender la naturaleza de manera activa, incorporando en ellas las raíces humanas de lo creativo, tal como el arte, la religión, la producción material y el amor, para trascender a la destrucción de la vida y de lo vivo.

Desde una mirada de salud mental basada en la satisfacción de necesidades humanas con referencia a los determinantes sociales^40^, es posible aseverar que una persona puede ver afectado su nivel de bienestar en la medida que los factores determinantes intrínsecos o extrínsecos colaboren o interfieran sobre su posibilidad de responder satisfactoriamente a una o más de estas necesidades. Así, el proceso de aculturación que enfrenta la población migrante toma relevancia y es comprendido como “el conjunto de transformaciones internas y conductuales experimentadas por una persona que está inmersa en una situación de contacto con una cultura diferente, con consecuentes cambios tanto en el individuo como en la cultura que lo acoge” y, en quien migra, obedecen a la búsqueda de sobrevivencia en un medio que le resulta nuevo y desconocido^41^. Por su parte, John W. Berry^42^ propone una definición de aculturación desde las actitudes de los sujetos migrantes hacia su cultura de origen y, por otro lado, desde las actitudes hacia la cultura predominante en destino^43^. Desde las combinaciones resultantes entre estas dimensiones, surgen cuatro orientaciones diferentes de la aculturación: la integración que describe a las personas que migran como interesadas por mantener su cultura de origen y también por mantener contacto con los miembros de la cultura predominante en la sociedad de destino, a la que la evidencia atribuye mejores resultados con relación a la salud mental de los migrantes. En segundo lugar, la estrategia de asimilación se dibuja desde la priorización de la interacción con la cultura predominante por sobre su cultura de origen. En tercer lugar, la separación se presenta como una estrategia propia de los sujetos que migran y privilegian el vínculo con su cultura de origen por sobre el interés en interactuar con los miembros de la cultura dominante y, finalmente, la marginación como estrategia presente en las personas migrantes que no muestran interés alguno en el vínculo con su propia cultura ni con la cultura dominante en destino^42^.

Al profundizar en este punto, distintos autores vinculan al proceso de aculturación en el contexto de migración con altos niveles de estrés psicológico, y cuyo resultado tiene relación directa con la adaptación o ajuste de la población migrante a la cultura del país anfitrión^43^. En este sentido, las determinantes sociales asociadas tanto a la cultura de origen, como a los espacios de subsistencia de las sociedades de destino repercuten directamente sobre el bienestar psicológico y en la integración social que los sujetos migrantes puedan alcanzar. Desde este lugar, dentro de las determinantes sociales incumbentes en la salud mental de la población migrantes se incluye a las propias características de las personas, tales como su nivel previo de salud mental, la exposición a estrés, la presencia de trastornos mentales previos, y sus estilos de apego, así como también la edad, el género, el nivel socioeconómico y educacional. Asimismo, entre los factores externos, destaca la relevancia de la ocupación, el contacto con los miembros de la cultura dominante, el dominio del idioma local, la expectativa de calidad de vida en el país de destino, y la discriminación percibida^39^; que al presentarse de manera frecuente, exponen a los sujetos migrantes a niveles elevados de estrés que terminan repercutiendo negativamente sobre su salud mental^44^. 

Desde el planteamiento de Fromm, estas fuentes de estrés ponen en juego la satisfacción de la necesidad de arraigo de la población migrante, dada la distancia con el lugar y la cultura de origen, así como por la existencia de obstáculos para establecer nuevos vínculos en la sociedad de destino, que también comprometen su autoconstrucción de identidad al percibirse como sujeto migrante distinto que representa la otredad cultural, fenotípica y de origen y que se erige como objeto de rechazo. Desde ese lugar, mientras mayor sean las divergencias existentes entre la cultura de origen y de destino, el proceso de aculturación ocurrirá de manera más tórpida, dadas las tensiones propias de dicha aculturación, que se asocia con un aumento en el riesgo de aparición de problemas de salud mental en la población migrante. Por otro lado, Tizón Grinberg y Grinberg -citado en Salvador^45^- define las ansiedades y defensas específicas que provoca cada una de las etapas del proceso migratorio:

Etapa 1: Frente a la decisión de migrar surgen ansiedad y culpa por lo que se ha decidido abandonar, manifestando defensas relacionadas a la negación de la pérdida.

Etapa 2: Al llegar a la sociedad de destino surgen ansiedades relacionadas con la confusión ante la dificultad de diferenciar los sentimientos entre lo que se ha dejado y lo nuevo. Aparecen ansiedades persecutorias frente a las exigencias del nuevo medio, como integrar el nuevo idioma y asegurar la subsistencia. En esta etapa, la ayuda recibida del medio externo contribuirá a la disminución de estas ansiedades, sin embargo, en ausencia de esta ayuda del medio, se incrementan las ansiedades paranoides, apareciendo mecanismos de defensa como la disociación, las idealizaciones compensatorias del país de origen y las identificaciones proyectivas, propias de un proceso de aculturación con resultados distintos a la integración.

Etapa 3: En esta etapa el duelo se logra elaborar a través de la integración a la cultura dominante, donde los sujetos migrantes renuncian a algunos acervos culturales propios para incorporar otras de la nueva cultura a la que se integran, de manera similar a los planteamientos de Berry^42^.

En complemento, González^46^ sostiene que para ningún duelo es recomendable el olvido, incluido el duelo migratorio, en tanto su elaboración se caracteriza por la integración de lo nuevo y la reubicación de lo dejado atrás, en un proceso complejo y no exento de dolor y de sufrimiento^46^. En este tipo de duelo, la pérdida tiene características más similares a una separación que a la muerte de un ser querido, dado que el objeto de la pérdida, que es complejo y multifactorial, prevalece en su existencia a lo largo del tiempo, puesto que los lazos con el país de origen se reactivan recurrentemente por distintos motivos, provocando también la reactivación recurrente del dolor experimentado asociado al duelo migratorio. 

Respecto a los objetos vinculados al duelo en migración, Achotegui^36^, establece que una de sus características es ser un duelo múltiple, y lo llama “los siete duelos de la migración” en consideración a la cantidad de cambios que experimenta de manera simultánea la persona que migra, cuya magnitud aumenta en la medida de la lejanía cultural entre el país de origen y el de destino. Dentro de los siete duelos, se identifica a la familia y los seres queridos como la separación más importante, en tanto afecta al apego de las personas. En este punto, destaca otro planteamiento de Achotegui^47^, que vincula íntimamente al duelo migratorio con las vivencias infantiles de quienes migran y la estructuración del apego, en tanto, la confianza básica es una de las vivencias de la infancia más tempranas y significativas en la manera en que se estructuran los lazos vinculares en los seres humanos^48^. En este primer duelo se pone en juego tanto la separación de los seres queridos, como el estrés asociado a tener que buscar nuevas relaciones vinculares en el país de destino. 

El segundo duelo tiene que ver con la lengua, el lenguaje y sus códigos, en que se considera la pérdida del contacto con la lengua materna junto con el esfuerzo de tener que adaptarse a la lengua y los códigos del país de acogida. El tercer duelo se refiere a la cultura con sus hábitos, costumbres y valores con el consiguiente estrés debido al alejamiento con la cultura de origen y el esfuerzo por conectar y adaptarse a la cultura del lugar de destino. En el cuarto duelo se consideran los elementos físicos de la tierra natal, como paisajes, colores, luz, aromas y temperatura, los que guardan una especial significancia a nivel afectivo para el que migra, junto con los efectos que tiene para la salud física y mental la exposición a determinada cantidad de luz natural. El quinto duelo tiene que ver con el estatus social, acceso a oportunidades, el uso y manejo de documentos, trabajo, vivienda y sistema de salud, y cómo esto afecta al nivel económico, el acceso a bienes culturales, a los servicios y atención, al ejercicio de ciudadanía y a la libertad. El sexto duelo se relaciona con el sentido de pertenencia, la percepción de ser parte de algún grupo de pertenencia, con una identidad determinada, la que se modifica debido a la migración, poniendo a la persona migrante en una situación en que corre un alto riesgo de volverse objeto de prejuicios y discriminación, conviviendo con la etiqueta de migrante mientras buscan un nuevo grupo de pertenencia. Y el séptimo duelo se refiere a la seguridad física y el riesgo para su integridad como persona, debido a la exposición a los numerosos cambios ambientales y los riesgos relacionados con el proceso migratorio^1^ que la persona se ve obligada a asumir por su condición de inmigrante, junto a la disminución de los factores protectores y recursos para sobreponerse a estos riesgos, fomentando una autopercepción de indefensión. 

Esta multiplicidad de aspectos podría dibujar repercusiones sobre la identidad de los sujetos migrantes, así como de la aparición de sensaciones de agobio, angustia e inseguridad durante la vivencia de las etapas de negación o resistencia frente al duelo, que pueden gatillar la existencia de cambios conductuales notables, tales como: actitudes regresivas, comportamientos sumisos y dependientes o la idealización de figuras de poder o episodios de quejas exacerbadas frente a situaciones frustrantes. Sin embargo, y como contrapunto, en una etapa de aceptación del duelo, quien migra puede tener la oportunidad de construir una identidad más rica y compleja, que le permita integrar lo bueno y lo malo tanto de su antigua como de su nueva realidad, en paralelismo con el concepto de integración frente a la aculturación descrito por Berry^42^. 

## MATERIALES Y MÉTODO

El diseño metodológico de esta investigación se fundamenta en el paradigma cualitativo, interpretativo y exploratorio a través de un proceso interactivo de construcción de la realidad entre investigador y participantes. Para su abordaje se plantea además un enfoque desde el interaccionismo simbólico con el fin de “identificar y comprender el significado de las acciones de las personas en la interacción humana dentro de su propio contexto social^” (^^49^.

Cabe señalar que estos resultados de investigación corresponden a una categoría de análisis emergente en el contexto de la investigación mayor titulada “Prácticas culturales vinculados al proceso de crianza de los hijos e hijas de madre migrante latinoamericana en Chile: una aproximación intercultural desde la cotidianidad”, cuyo grupo de estudio fue conformado por 39 madres, de las cuales 19 eran venezolanas, 11 peruanas y 9 haitianas, seleccionadas a través de muestreo teórico intencionado y que cumplieron con los criterios de inclusión definidos en razón del constructo teórico y objeto de estudio de esta investigación mayor, correspondientes a: residir en comunas de alta concentración de población migrante^8^^,^^14^^,^^50^; que se encuentren en proceso de crianza de hijos o hijas menores de 4 años, que hayan nacido o no en Chile; que sean personas usuarias del nivel primario público de salud; que sus hijas e hijos asistan a la educación preescolar; y que hayan manifestado su intención de participar en la investigación a través de la firma de consentimiento informado aprobado por el Comité de Ética institucional de la Universidad de Santiago de Chile según consta en Informe Ético No. 454/2021. 

Para aproximarse a las madres migrantes participantes, se tomó contacto con los jardines infantiles de mayor matrícula migrante de las comunas de Santiago, Independencia y Estación Central debido a la concentración de población migrante en estas zonas de la Región Metropolitana^8^. Luego, se estableció contacto telefónico y/o presencial con las madres para explicarles el objetivo de la investigación e invitarlas a participar, así como solicitarles la firma de consentimiento informado para formalizar una entrevista posterior.

Como técnica de recolección de datos se realizaron entrevistas semiestructuradas, de aproximadamente una hora de duración, entre los meses de octubre de 2021 y enero 2022, vía telemática, debido a la emergencia sanitaria por Sars-cov-2. Se contó con apoyo de una mediadora intercultural no profesional^51^ para la realización de entrevistas a las madres migrantes haitianas participantes. Cada una de las 39 entrevistas realizadas fueron transcritas y analizadas manualmente mediante la técnica de análisis de contenido temático^45^ y fueron identificadas con la inicial del término entrevistada (E), seguida de la inicial de su nacionalidad y por el numero correlativo de la entrevista (por ejemplo: EP1).

La saturación de la información se consiguió una vez que los relatos obtenidos no aportaron nuevos antecedentes vinculados a las categorías de análisis definidas ni otros hallazgos de interés para la investigación mayor, que se relacionaran con la crianza de los hijos e hijas de madres migrantes en Chile. En el desarrollo de dichas entrevistas afloraron de manera emergente los relatos que dieron cuenta sobre los alcances emocionales en la salud mental de estas madres migrantes, relacionados con la crianza en situación de migración, los que fueron objetivados en la medida que se realizaron las entrevistas y también desde su posterior análisis. 

Cada una de las entrevistas fue leída una y otra vez para, según Morse y Field^52^, “identificar significados y alcanzar su comprensión más profunda y hacer evidente lo invisible” en un permanente proceso de conjetura, corrección, modificación, sugerencia y defensa de los datos y sus relaciones. Los datos disponibles fueron revisados en función de su contenido y, a su vez, reducidos y compactados para su cotejo con los datos reales y los definidos *a priori* para la investigación en sus objetivos específicos. A través de esta codificación abierta, los datos se vincularon a conceptos ya identificados o subyacentes para dar forma a categorías y subcategorías de análisis en un menor grado de abstracción, según Polit y Hungler^53^. En un siguiente nivel de análisis, se reagruparon los datos obtenidos a partir de la codificación para precisar los argumentos en relación con el fenómeno en estudio. De este modo, y a través de la comparación constante de las codificaciones en búsqueda de similitudes y diferencias, se reconstruyeron los datos para identificar patrones, secuencias y relaciones, con lo que se dio forma a categorías y subcategorías de análisis^53^ que permitieron afinar las categorías teóricas para llevar posteriormente el análisis a un nivel mayor de abstracción, según Corbin y Strauss^54^. Es así como, desde la teorización de los resultados obtenidos, se obtuvieron tres categorías de análisis derivadas de la pregunta de investigación^53^: 1) la pérdida de los vínculos y la lejanía con el origen, 2) expectativas versus realidad y 3) validando sobreesfuerzos.

## RESULTADOS

### La pérdida de los vínculos y la lejanía con el origen

Los relatos de las madres migrantes entrevistadas dejan entrever expresiones de profunda tristeza en relación a las perdidas vivenciadas, producto de su proceso migratorio, atribuyendo incluso la connotación de una experiencia indeseable de vivir por cualquier ser humano, dado el dolor implícito en esta experiencia, el cual se exacerba producto de las presiones propias del proceso de integración social y de la subsistencia en destino en un diario desarraigo. Son referidas por las entrevistadas emociones de tristeza, inseguridad, decepción, impotencia y soledad. Estas experiencias de dolor y pena muestran una afectación de la salud mental desde la insatisfacción de las necesidades de arraigo y vínculo propuestas por Fromm^38^, asociadas a la pérdida por la lejanía con el lugar de origen, con las relaciones vinculares significativas, y la percepción de pérdida del soporte social, vinculable al duelo por la seguridad física propuesto por Achotegui^1^. Desde ese lugar, los resultados obtenidos arrojaron que la referencia a estos sentimientos es compartida por las madres venezolanas, peruanas y haitianas entrevistadas.

*…Yo creo que la maternidad es una de las cosas más difíciles, y yo creo que ser mamá y además migrante, no es fácil, o sea, es una carga emocional mucho más fuerte porque al menos en Venezuela una tiene el apoyo* [llora] *de tu mamá, de tu papá, aquí el hecho de tener que pagar arriendo no es fácil, o sea, yo pago lo de arriendo lo que yo cobro de sueldo.* (EV5)

*…Una reflexión: quisiera que de verdad nadie tuviera que pasar por una migración, que nadie emigrara de su país* [llora] *porque no quiero que nadie tenga que pasar por esto. Una cosa es migrar por disfrute porque quiero irme a otro país, a que lo tengas que hacer por obligación, no es fácil.* (EV2)

*…Lo que he aprendido en este país como mamá es la impotencia, porque como te explico, a veces hay que tragar grueso.* (EV11)

*…Mira, no es fácil porque por decirlo prácticamente estoy sola, y ahora estoy pasando por una etapa de, como de que estoy con una especie de depresión, porque mi mamá falleció en abril y yo no pude viajar… Así que estoy con tratamiento por ataques de pánico y ansiedad, pero bastante bien sobre llevándolo.* (EV13)

*…Yo sí necesité una psicóloga porque me dio como depresión, porque me sentía muy sensible, todo era llorar y llorar, me sentía vulnerable a cualquier cosa.* (EP10)

*…Acá en Chile para criar un niño es muy difícil, porque no tengo a nadie y a veces las cosas son complicadas, pero con amor y paciencia lo hago todo, con amor lo hago todo por mi hijo*. (EH1)

La nostalgia, el dolor y la angustia se transforman en expresión de síntomas depresivos que cursan las madres migrantes^55^ con efectos y secuelas en su salud física y mental producto del proceso migratorio^56^, lo que es interpretable como una de las reacciones esperables producto de la complicación del duelo migratorio experimentada por las madres migrantes dentro del espectro del “síndrome de Ulises”^36^, tales como: la disminución del apetito, la fatiga y malestar inespecífico^49^. Estas consecuencias se hacen extensivas a sus hijos e hijas por cuanto existe evidencia que indica que las madres migrantes que cursan depresión pueden desencadenar alteraciones socioemocionales en sus hijos e hijas, comparables a las que tienen madres no migrantes^55^.

*…Sí, o sea, yo soy baja, de estatura, yo no debo pesar mucho porque soy baja, pero yo estaba en el límite, yo estaba pesando 43 kilos, porque mi peso es entre 45 y 50 kilos. Yo no sé si eso pudo haber afectado al embarazo, no sé, preocupación quizá, estrés, yo digo que quizá eso pudo ser, porque también a mi pareja le dio una parálisis facial, en la cara, y él estaba con terapia y se nos iba prácticamente todo el dinero, lo poco que ganamos.* (EV6)

*…En algún momento estuve depresiva, muy depresiva, fue terrible, fue un caos mi casa, porque en verdad, mi esposo no se daba cuenta, pero yo le decía me siento mal, y yo soy hipertensa, entonces comencé a ver luces de la misma desesperación.* (EP2)

### Expectativas versus realidad

Como ya se estableció, sentimientos y expresiones de frustración estuvieron presentes en numerosos relatos de las madres migrantes entrevistadas, fundados en la imposibilidad de cumplir con las expectativas y deseos que cimentaron su proyecto migratorio con relación al nivel socioeconómico alcanzado en destino y la posibilidad de criar a sus hijos e hijas en coherencia con estos. Los sentimientos se profundizan en un contexto social en el cual, alejadas de su cultura de origen y sus redes de apoyo, las madres experimentan frustración por no poder acceder a apoyos del medio externo en el proceso de migrar, comprometiendo así su ideal acerca del ejercicio de su rol materno sustentado en sus aspiraciones y en sus propios capitales culturales. La realidad encontrada al llegar al país de acogida es notoriamente distante de la esperada, y las pone en una posición de sobrevivencia que les obliga a negociar este ideal acerca de la maternidad, teniendo que priorizar su rol de madre proveedora por sobre su rol reproductivo y de madre cuidadora y criadora.

*…Mire, criar un hijo es difícil pero como migrante tiene sus dificultades adicionales. Primero, en mi caso es criarlo en un entorno ajeno a ti, sentir quizá que no tienes la confianza del entorno, porque no lo conoces, no sentirte quizá reconocida*. (EV11)

*…Yo tengo que trabajar para ayudar a mi hijo para ir a la escuela, como mamá migrante no es fácil porque tiene que trabajar para ayudar a los niños, para conseguir todo lo que necesita y para que pueda continuar sus estudios hasta que lo necesite, por eso tenemos que trabajar para cumplir con las necesidades de mi hijo.* (EH7)

Así, se configura una representación de madre que comparte la satisfacción que el trabajo remunerado significa para su rol de proveedora, pero que al mismo tiempo experimenta el duelo por pérdida de estatus social alcanzado en su país de origen. Así, las madres migrantes entrevistadas, conscientes de su condición de migrantes en un medio socialmente demandante, que a la vez no termina de responder a sus expectativas de apoyo, sufren ante la imposibilidad de dedicar el mayor tiempo posible a la crianza de sus hijos e hijas debido a extensas jornadas laborales, pero que soportan para satisfacer las necesidades básicas de sus hijos e hijas. Frente a estas vicisitudes, las madres migrantes entrevistadas delegan el cuidado de sus hijos e hijas durante sus jornadas laborales, lo cual constituye un motivo de frustración, en tanto reconocen como dolorosa la acción de delegar su cuidado, pero que resignifican como un mal necesario que cotidianamente tensiona sus expectativas de criar en una sociedad que les permite la subsistencia y, a la vez, fortalecer el vínculo madre hijo o hija.

Cabe señalar que para el caso de algunas de las madres haitianas entrevistadas, estas aprehensiones se trasladan a sus lugares de trabajo -informales y de comercio ambulante- a los que estas madres deben asistir en compañía de sus hijos e hijas ante la ausencia de redes de apoyo, familiares, connacionales e institucionales para el cuidado de sus hijos e hijas. Esta condición acrecienta su indefensión y vulnerabilidad de sus espacios de subsistencia en el país, instalando una doble presencia entre cuidado y trabajo que acrecienta el duelo vivenciado, dada la pérdida de la sensación de seguridad física de estas madres configurando en sí un círculo perverso.

*…a mi hijo, otra persona lo está criando.* (EP11)

*…Ay, la verdad, criar un hijo aquí siendo migrante es fuerte, porque uno tiene que, a la vez, atender, trabajar, es duro. Después, fue que me puse a hacer mi propio negocio: esos helados que venden de fruta. Yo los míos los preparaba y me iba con mi hija en el coche. Ella estaba bebecita y me la llevaba hasta la feria, a todas las ferias que había en la semana. A todas las ferias que había yo me iba con ella. Después hasta vendí desayunos, me iba a migración a vender desayuno, la vi aquí muy duro para tener hijo, súper duro.* (EH4)

### Validando sobreesfuerzos

Sin embargo, frente a los sufrimientos experimentados y los esfuerzos realizados por las madres migrantes para subsistir en migración, varias de ellas aportan relatos que dan cuenta de un deseo inherente de surgir a través del sacrificio y que resignifican como un camino legítimo para alcanzar la vida mejor y evitar la reproducción de experiencias carentes, presentes en las biografías de las madres migrantes entrevistadas. Con miras de futuro, destaca en las madres migrantes haitianas la relevancia entregada al ahorro y la superación propia a través del estudio y el trabajo, para asegurar la consecución de bienes materiales para la subsistencia.

*…Es difícil, pero se puede. O sea, es duro, pero una parte compensa a la otra. Igual es complicado como mamá migrante soltera, pero sí se puede.* (EV17)

*…Mi sueño es tener un diploma para trabajar y ayudar más a mis hijos… no quiero que mis hijos pasen lo mismo que yo he pasado, por eso quiero seguir estudiando, para vivir y luchar y que mis hijos tengan otra vida y no la que yo tenía.* (EH3)

*…Chile es un gran país, que te da la oportunidad de seguir, pero lo importante es que uno tiene que tener la cabeza muy plena y estudiar, porque cuando tú vienes a un nuevo país, hay que estudiar, aprender la lengua para salir adelante es la primera cosa. Y la segunda es ir a la escuela y tercero, que si usted no tiene ninguna profesión tiene que luchar, tiene que comer menos para pagar algún técnico o peluquería o cualquier cosa*. (EH7)

*…Tiene que tener preparación, para vivir, para comer. Si un amigo tiene algo y el niño quiere, también tengo que comprárselo a mi hijo. Y también tengo que prepararme para que mi hijo pueda estudiar en el futuro, ahorrar algo para eso*. (EH9)

### DISCUSIÓN

Para las madres migrantes entrevistadas, la crianza de sus hijos e hijas en un contexto de migración ha significado una experiencia difícil. En sus relatos ellas anteponen la resignificación de sus procesos migratorios desde una experiencia impulsada por el deseo de alcanzar mejoras en su subsistencia, pero que, por otro lado, constituye un camino pavimentado por pérdidas a distintos niveles, sufrimientos, angustias, sentimientos de desesperanza y desarraigo: desde su mirada, un camino necesario para alcanzar la ansiada vida mejor. En los relatos obtenidos de las madres migrantes entrevistadas se dejan entrever expresiones de profunda tristeza en relación con las pérdidas vivenciadas en su proceso migratorio, atribuyendo incluso la connotación de una experiencia indeseable de vivir por cualquier ser humano, dado el dolor implícito en esta experiencia, el cual se exacerba producto de las presiones propias de la búsqueda de integración social y la subsistencia en destino en un diario desarraigo. Este hallazgo se refrenda con los resultados de una investigación de Millaleo^55^, realizada con mujeres peruanas migrantes en Chile, quienes ya anteriormente han expuesto experiencias migratorias llenas de amargura, soledad, tristeza y encierro. 

Existen además algunos relatos aportados por mujeres migrantes en algunas investigaciones de Bonhomme^57^ en los que comparten el dolor y la tristeza a diario una vez llegadas al país y que se relacionan estrechamente con la fase de resistencia en el duelo migratorio. La evidencia existente da cuenta de la valoración de los primeros meses de migración como muy tristes, dominados por el llanto, y en donde el deseo de regresar a su país de origen se convierte en un protagónico deseo^57^. Siguiendo este planteamiento, otros antecedentes aportados por una investigación realizada por Gallardo^28^, con relación al estrés parental observado en población migrante en Chile, indican que las madres migrantes que llevan menos tiempo viviendo en el país, presentan un mayor nivel de estrés, dada la resistencia en el contexto del duelo migratorio, que se hace más evidente mientras menor sea la edad de sus hijos e hijas^28^, lo que puede guardar relación con la fragilidad y las necesidades de cuidado propias de los niños a edades más tempranas.

Para las madres entrevistadas, la migración constituye un sinónimo de esperanza para optar a nuevas oportunidades, pero que implican un particular esfuerzo. Si bien la migración constituye una esperanza de solución frente a las dificultades de vida de las mujeres migrantes en sus países de origen, también encierra un lado oscuro o controversial denominado estrés o duelo migratorio. En ocasiones y ante la imposibilidad de cumplir sus expectativas frente a la migración en un medio altamente exigente y demandante, las madres migrantes entrevistadas alojan sentimientos de victimización y culpa, al mismo tiempo que son embargadas por la angustia y la impotencia al ver cómo la agencia en la crianza de sus hijos e hijas se compromete en una sociedad que esfuma sus esperanzas de entregar a sus hijos e hijas la ansiada vida mejor gozando de su diaria compañía. Siguiendo a Byung-Chul Han^58^, la sociedad actual es una sociedad de alto rendimiento que se especializa en la fabricación de individuos depresivos y fracasados los que, al no dar la talla de las exigencias para sobrevivir, caen en autoexplotación, autotormento y extenuación que, al mismo tiempo, es causa de su fracaso al conformar en sí mismo una suerte de círculo perverso^58^. 

La experiencia de migrar y de criar sus hijos en un lugar extraño de sus redes de apoyo ha significado, para algunas de las madres migrantes entrevistadas, enfrentar un duelo específico frente a la pérdida de la construcción del ideal de maternidad según su cultura de origen, por cuanto la precariedad y devaluación de sus nuevas condiciones de vida tensionan su ideal de crianza, desencadenando consecuencias objetivas en su propia salud física y psicológica^59^. La priorización de la función productiva por sobre la reproductiva privilegia la supervivencia y, de este modo, la autoexigencia provoca en las madres migrantes entrevistadas sentimientos de frustración y vergüenza de su insuficiencia, que las despoja de cualquier vestigio de autoestima, justificando su infortunio y humillación^60^. Así, se mantienen vivas las ansiedades en torno a la migración, obstaculizando su integración en una permanente alerta frente a la lucha por la sobrevivencia^61^, aumentando las posibilidades de transformar este duelo migratorio en un estrés de tipo crónico: mientras mayores sean los factores estresores medioambientales en que viva la población migrante, más difícil será elaborar el duelo migratorio, profundizando así su vulnerabilidad^57^. Siguiendo los planteamientos de González^46^, en las madres migrantes participantes de este estudio se identificaron, como predictores de un duelo migratorio complicado, la dificultad para establecer un ritmo de vida normalizado, sentimientos de soledad, miedo y fracaso, y la percepción de tener que luchar para sobrevivir por estar sometido a condiciones de explotación o humillación^47^. 

La complejidad que arrastra el duelo migratorio se sostiene en que la experiencia de migrar involucra su mundo, en cuyo duelo se entretejen distintas pérdidas y, para afrontar dicha pérdida, los migrantes atraviesan su proceso de duelo al mismo tiempo que tratan de sobrevivir e integrarse al nuevo ambiente. En este proceso vivencian distintas y difíciles instancias de integración, contextualizadas en frágiles circunstancias personales y sociales que incluso pueden llegar a desestructurar sus vidas y que se vinculan a la pérdida del estatus social en el país de origen, sus redes sociales y el alejamiento de espacios de representación e identidad, dejando huellas físicas y afectivas^62^. Además, la discriminación y el desarraigo del que son víctimas acentúan sus tristes experiencias y dificultan su integración a través del trabajo y la socialización, para tejer nuevas redes sociales en el país profundizando así su vulnerabilidad y sus autopercepciones de abandono y desprotección, por cuanto restringen su posibilidad de abrir nuevos universos culturales y facilitar así su integración^53^. Al profundizar desde la lógica del duelo migratorio como un duelo múltiple en relación a “los siete duelos” propuestos por Achotegui^47^, en los relatos de las madres entrevistadas presentan mayor relevancia las pérdidas asociadas a los vínculos y redes de soporte social, la percepción de seguridad y el estatus social, con especial mención en el duelo que enfrentan de su propia cultura^1^, y desde la que proviene el imaginario de un estilo de maternidad ideal traído desde sus países de origen y que se ven en la obligación de postergar o renunciar en pro de la priorización de la subsistencia. 

Sin embargo, frente a estas vicisitudes, las madres migrantes entrevistadas exponen permanentemente en sus relatos la fuerza motivadora que les inyecta el espíritu de superación para soportar las dolorosas experiencias relatadas. Para hacer frente a la pena y frustración producto del desarraigo y las frágiles condiciones de vida en las que intentan integrarse al país, las madres migrantes entrevistadas despliegan estrategias de negación de aquello que los aleja de la sociedad de destino, incorporando y también exacerbando prácticas y actitudes socialmente valoradas como el esfuerzo, la honradez y la voluntad de surgir con la intención de revertir el rechazo y estigmatización en la sociedad de destino y que además transmiten a sus hijos e hijas^63^. Estas prácticas han sido también identificadas en una investigación de Millaleo^55^, en población migrante peruana, en la que los discursos dejan huella sobre la alta valoración del esfuerzo sobrehumano para migrar, y el respeto a una sólida base valórica, como predictor del respeto al nivel socioeconómico alcanzado en Chile producto del propio esfuerzo^57^, que podría ser interpretado como un mecanismo de defensa, sustentado en la justificación de dicho esfuerzo (o sacrificio), que refuerza la identidad de madre, y también sirve de camino válido para cumplir con las expectativas de mejora social puestas en el acto de migrar^36^. De este modo, las madres migrantes entrevistadas construyen sobre ellas mismas una representación de “buenos migrantes” que se sustenta en el orgullo que significa para ellas proveer de los necesario para que sus hijos alcancen una calidad de vida y un nivel educacional mejor al que ellas vivenciaron y la satisfacción que implica el responder a los deseos y necesidades materiales de sus hijos e hijas argumentado desde el *“si uno quiere, puede”* (EV15).

## CONCLUSIÓN

Las madres entrevistadas refieren experiencias de duelo migratorio en que se vislumbran distintos matices según el país de origen. Sin embargo, la expresión de este duelo múltiple relacionada con el desafío de criar a sus hijos e hijas en un país extraño, en ausencia de redes de apoyo y de soporte social, que exige extenuantes esfuerzos para el sustento, las presiona a renunciar a su cosmovisión y expectativas frente a la maternidad, y que con dolor reconfiguran y supeditan al rol de madre proveedora por sobre el rol de madre presente. Estos duelos se manifiestan cotidianamente en expresiones de tristeza, en las que el desarraigo, la distancia con aquello que les ofrecía seguridad, la coherencia con su propia cultura, los vínculos afectivos y el soporte social, constituyen un denominador común de experiencias de integración que se tensionan con las expectativas gestadas en su proyecto migratorio, validando sobreesfuerzos físicos para la consecución de la vida mejor en un espacio de subsistencia especialmente adverso. Lamentablemente, estas tensiones obstaculizan la elaboración del duelo migratorio de las madres migrantes participantes, comprometiendo así su salud física y mental y perpetuando la vulnerabilidad de este grupo humano, en concordancia con la evidencia internacional disponible. En aquellos relatos que dieron cuenta de sintomatología concordante con el síndrome de Ulises, queda en evidencia la relación entre los estresores psicosociales, parte del proceso migratorio y los síntomas presentados, generando además una situación que recrudece las condiciones adversas que rodean a las familias inmigrantes, complicando aún más la resolución del duelo. Desde una lógica en favor de la participación y el empoderamiento comunitario, la implementación de estrategias desde los gobiernos locales que promuevan la conformación de grupos de apoyo para madres y familias migrantes tendría el potencial de mantener a las madres migrantes cercanas a su cultura originaria, facilitando el dialogo intercultural con la sociedad de destino. Estas estrategias con efectos sobre el sufrimiento por separación tributarían al logro de la integración social como resultado ideal del proceso de aculturación propuesto por Berry^42^.
